# Broadband nonreciprocal thermal emissivity and absorptivity

**DOI:** 10.1038/s41377-024-01520-3

**Published:** 2024-07-24

**Authors:** Komron J. Shayegan, Jae S. Hwang, Bo Zhao, Aaswath P. Raman, Harry A. Atwater

**Affiliations:** 1 Thomas J. Watson Laboratory of Applied Physics, California Institute of Technology, Pasadena, CA USA; 2 Department of Electrical Engineering, California Institute of Technology, Pasadena, CA USA; 3 Department of Materials Science and Engineering, University of California, Los Angeles, Los Angeles, CA USA; 4 Department of Mechanical Engineering, University of Houston, Houston, TX USA

**Keywords:** Metamaterials, Nanophotonics and plasmonics

## Abstract

A body that violates Kirchhoff’s law of thermal radiation exhibits an inequality in its spectral directional absorptivity and emissivity. Achieving such an inequality is of fundamental interest as well as a prerequisite for achieving thermodynamic limits in photonic energy conversion^1^ and radiative cooling^2^. Thus far, inequalities in the spectral directional emissivity and absorptivity have been limited to narrow spectral resonances^3^, or wavelengths well beyond the infrared regime^4^. Bridging the gap from basic demonstrations to practical applications requires control over a broad spectral range of the unequal spectral directional absorptivity and emissivity. In this work, we demonstrate broadband nonreciprocal thermal emissivity and absorptivity by measuring the thermal emissivity and absorptivity of gradient epsilon-near-zero InAs layers of subwavelength thicknesses (50 nm and 150 nm) with an external magnetic field. The effect occurs in a spectral range (12.5–16 μm) that overlaps with the infrared transparency window and is observed at moderate (1 T) magnetic fields.

## Abstract (web summary)

Nonreciprocal thermal absorptivity and emissivity is shown over a broadband spectrum and specified angular range for a gradient epsilon-near-zero photonic structure.

## Graphical abstract

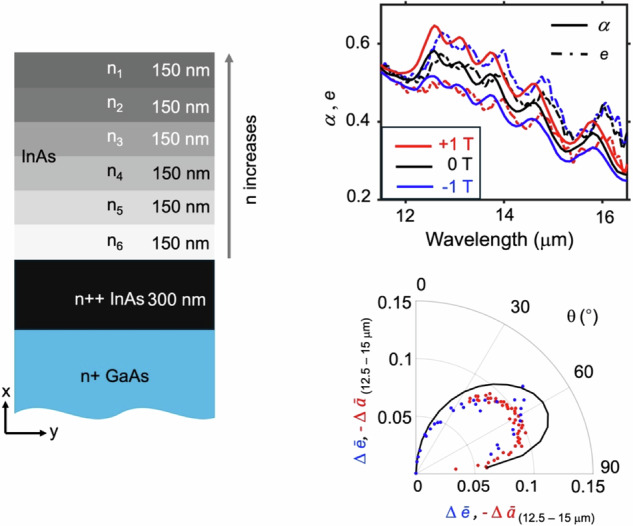

## Introduction

Nonreciprocal photonic elements constitute a basic building block of free-space^5^ and on-chip^6^ optical systems where isolation is required. Applications in microwave and optical communications have been realized, with methods of achieving nonreciprocal effects ranging from traditional magneto-optical effects to space-time modulation^7^. Recently, there has been an effort to realize nonreciprocal photonic structures in the context of thermal emission, that is, make a structure whose spectral directional emissivity and absorptivity are not equal^2,8^.

While still a nascent field, there have been recent demonstrations of narrowband nonreciprocal thermal emissivity and absorptivity in the infrared^3^ and nonreciprocal absorptivity in the long-wavelength infrared^9^. This report^3^ of a nonreciprocal effect relied on tuning the coupling of a guided-mode resonance with a plasma resonance, limiting the bandwidth and angular range of the absorptivity and emissivity detuning. Broadband nonreciprocal absorption at a single angle of θ = 60° was reported without direct measurement of emissivity^9^, using a graded index epsilon-near-zero (ENZ) structure.

In this paper, we carry out direct emissivity and absorptivity measurements on a gradient ENZ structure of subwavelength InAs layers in an external magnetic field and observe opposite tuning of the emissivity and absorptivity for the same directional channels. We carry out these direct measurements of both the absorptivity and the emissivity for two samples with different thicknesses and find that the nonreciprocal emission and absorption strongly depends on the sample’s subwavelength thickness and the carrier concentration ordering.

The ENZ resonance wavelengths are set by the electron free carrier concentration of each individual layer. We focus on results obtained from a gradient-ENZ structure with six carrier concentrations (n_1_ – n_6_) increasing from 1.5 to 4.5 × 10^18^ cm^-3^ from the bottom to the top of the structure^10^. The individual resonances of the layers are spectrally closely spaced to create a broadband emissivity and absorptivity feature in the 12.5 μm – 17 μm region. The angle at which the emitted light couples out of the structure is determined by the thicknesses of the constituent ENZ layers. This is formally known as the Berreman mode.

The mechanisms that determine the emitter spectral and directional selectivity (i.e., the carrier concentration and thickness of the constituent layers, respectively) also determine the bandwidth, directionality, and magnitude of the nonreciprocal tuning of the emissivity and absorptivity. Consequently, the contrast between low emissivity angles at near-normal incidence and high emissivity at large angles in addition to low emissivity outside versus high emissivity inside the ENZ bandwidth is tunable with the magnetic field.

**Fig. 1:**
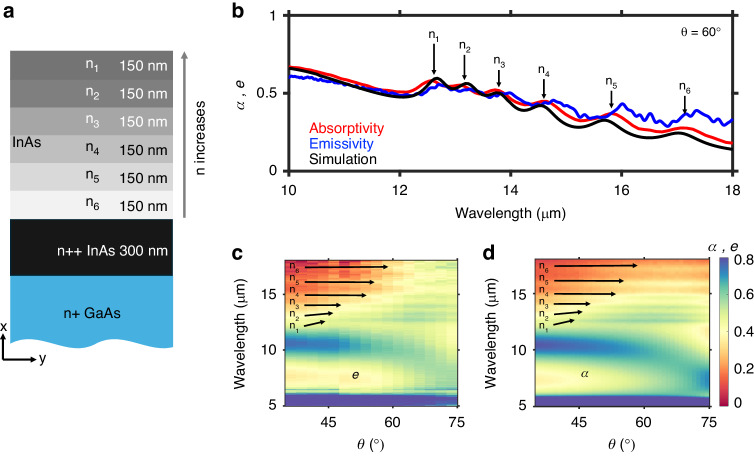
Schematic of device with absorptivity and emissivity measurements in the absence of an external magnetic field. **a** Schematic of the gradient-ENZ structure. The carrier concentration increases for layers closer to the surface of the device (i.e., in the increasing x-direction). A very degenerately doped n^++^ InAs layer is used as a back reflector. The back reflector layer thickness is to scale. **b** Absorptivity (red), emissivity (blue), and simulated (black) spectra for the structure at θ = 60°. The resonant absorption/emission associated with each layer are labeled. **c** Spectral directional emissivity data taken with the sample heated to 100 °C. **d** Spectral directional absorptivity data taken at ambient (25 °C). Full spectral directional data (including simulations) are included in Fig. S1

The thickness of each layer with distinct carrier concentration is 150 nm thick and is capped at the back with a 300 nm degenerately doped n^++^ InAs layer that acts as a back reflector (Fig. 1(a)). We also measure a structure with 50 nm thick individual layers, which is discussed later in the paper. Both structures are grown using molecular beam epitaxy on a GaAs [100] handle wafer.

## Results

We obtain the spectral directional emissivity and absorptivity of the sample using two different measurement systems (Fig. 1(b-d)). The absorptivity data is taken with a J.A. Woollam IR ellipsometer while the emissivity data is taken using a homebuilt angle-resolved thermal emission spectroscopy setup^11^. Comparing the absorptivity and emissivity spectra to simulations at the expected angle for maximum magnetic-field tuning (θ = 60°), we resolve the resonances that collectively form the broadband absorptivity and emissivity spanning 12.5–17 μm (Fig. 1b). The background slope from high to low emissivity and absorptivity from short (10 μm) to long (19 μm) wavelengths is attributed to absorption of the GaAs handle on which the sample is grown.

The absorptivity measurements are taken at room temperature (25 °C) while the emissivity measurements are taken with the sample heated to 100 °C. Heating the sample to higher temperatures results in a larger signal-to-noise ratio for emissivity measurements, however results in a slight redshift of the resonances associated with the ENZ-crossings (Fig. 1b, blue trace). Comparing the full spectral and angular information acquired from emissivity and absorptivity measurements, we see that the qualitative agreement between the two measurements matches across a large angular range (35° < θ < 75°) (Fig. 1(c–d), Fig. S1). There are benefits to the two measurement systems: the emissivity setup can access the near-normal angular range of the sample emission while the absorptivity setup has greater accuracy and precision in the individual angle certainty owing to it not being a homebuilt setup or requiring a lens for collection. There is increased signal uncertainty at longer wavelengths ( > 16 μm) in the emissivity measurement setup due to both the polarizer transmission function and the detector sensitivity at these wavelengths. A more in-depth description of the emissivity and absorptivity measurements is included in the Supplement.

The intensity of the resonances corresponding to n_1_–n_6_ have a strong angular dependence, however the spectral position of the resonance is constant as a function of angle, indicative of a distinctive broadband leaky Berreman mode supported by gradient-ENZ materials^12,13^. This simplifies our analysis of the magnetic-field effect on the emissivity and absorptivity tuning in that we can average over a fixed broadband spectral range and look at the angular dependence of the tuning without losing the spectral range where the effect is occurring.

When we apply a magnetic field, the permittivity scalars of the ENZ layers turn into a series of anti-symmetric tensors:

E1 $${\varepsilon }_{i}\left(\omega \right)=\,\left[\begin{array}{ccc}{\varepsilon }_{{\rm{xx}}} & {\varepsilon }_{{\rm{xy}}} & 0\\ {\varepsilon }_{{\rm{yx}}} & {\varepsilon }_{{\rm{yy}}} & 0\\ 0 & 0 & {\varepsilon }_{{\rm{xx}}}\end{array}\right]$$

E2 $${\varepsilon }_{{\rm{xx}}}={\varepsilon }_{{\rm{yy}}}={\varepsilon }_{\infty }-\frac{{\omega }_{{\rm{p}},i}^{2}\left(\omega +i{\Gamma }_{i}\right)}{\omega \left[{\left(\omega +i{\Gamma }_{i}\right)}^{2}-{\omega }_{{\rm{c}},i}^{2}\right]}$$

E3 $${\varepsilon }_{{\rm{xy}}}=-{\varepsilon }_{{\rm{yx}}}=i\frac{{\omega }_{{\rm{p}},i}^{2}{\omega }_{{\rm{c}},i}}{\omega \left[{\left(\omega +i{\Gamma }_{i}\right)}^{2}-{\omega }_{{\rm{c}},i}^{2}\right]}$$

E4 $${\varepsilon }_{{\rm{zz}}}={\varepsilon }_{\infty }-\frac{{\omega }_{{\rm{p}},i}^{2}}{\omega \left(\omega +i{\Gamma }_{i}\right)}$$

where ${\omega }_{{\rm{p}},i}=\sqrt{{n}_{i}{e}^{2}/({m}_{{\rm{e}},i}{\varepsilon }_{0})}$ and ${\omega }_{{\rm{c}},i}={eB}/{m}_{{\rm{e}},i}$ are the plasma and cyclotron frequencies for each layer, *i*. The free carrier density *n*_*i*_, effective mass *m*_e,*i*_ and scattering rate Γ_*i*_ used in the simulations are included in Table 1. The external magnetic field effects only transverse-magnetic (TM), or p-polarized emission and absorption in this configuration^14,15^.

**Table 1:** Material properties in simulations

Layer	carrier concentration (10^18 ^cm^-3^)	m_e_ (9.11 × 10^-31^kg)	Γ (THz)
n_1_	4.5	0.053	5.653
n_2_	3.9	0.050	5.652
n_3_	3.3	0.046	5.651
n_4_	2.7	0.042	5.650
n_5_	2.1	0.038	5.649
n_6_	1.5	0.032	5.649
n_reflector_	60	0.158	10.06
n_GaAs_	2.1 × 10^-12^	0.063	3.2824

**Fig. 2:**
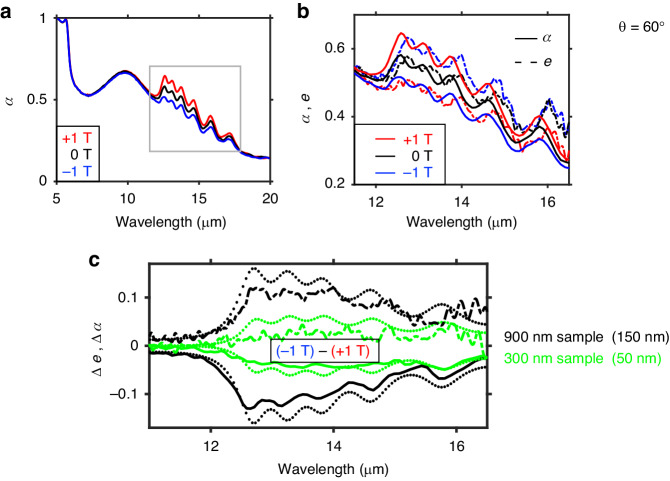
Nonreciprocal absorptivity and emissivity measurements for a + 1 T and - 1 T applied magnetic field. **a** Absorptivity data taken at θ = 60° for varying magnetic field values. The resonant peak near 6 μm is the n^++^ back reflector. The light gray box highlights the broadband spectral range where the tuning is observed. **b** Absorptivity and emissivity plots zoomed in to the spectral range of interest. The opposite magnetic field dependence is observed in the emissivity data. We note that the emissivity data is noisier at longer wavelengths where detector sensitivity is reduced. **c** Simulated (dotted) and measured Δ *e* (dot-dashed) and Δ *a* (solid) spectra for θ = 60° when subtracting the + 1 T measurement from the – 1 T measurement. The thick sample (black) shows a stronger and spectrally inhomogeneous tuning when compared to the thin sample (green)

The measured absorptivity spectra at θ = 60° for an applied field of – 1 T, 0 T, and + 1 T are shown in Fig. 2a. We observe tuning across the entire wavelength range (12.5–17 μm), with stronger tuning at shorter wavelengths. This nonuniformity in the spectral tuning is a consequence of the ordering of the layers. As discussed later in the paper (Fig. S6 and S7) the ordering and thicknesses of the layers constituting the gradient-ENZ sample can be judiciously chosen to achieve uniform spectral tuning.

We compare the magnetic field dependence of the absorptivity to the emissivity of the sample under the same applied magnetic field strengths (Fig. 2b, Fig. S2). The opposite magnetic field dependence of the emissivity of the sample demonstrates nonreciprocal broadband absorptivity and emissivity, and implies a violation of the spectral-directional Kirchhoff thermal radiation law^3^. We note that the emissivity spectral features are slightly red-shifted from the heating of the sample, and that the strong match between the zero-field (Fig. 2b, black traces) absorptivity and emissivity data degrades slightly at long wavelengths. This is a consequence of detector sensitivity at long wavelengths (λ > 16 μm) decreasing in the emissivity setup. Despite the low emission collected from the sample at these wavelengths, the detected emission from the reference blackbody is also low, resulting in a normalization that is increasingly noise sensitive.

We also measure the difference in the emissivity and absorptivity (Δ *e*, Δ *a*) from - 1 T to + 1 T for a second sample with the same gradient carrier concentration profile but with individual layer thicknesses of 50 nm (Fig. 2c). The Δ *e* and Δ *a* for this second, thinner sample is notably smaller than what is observed for the thicker sample and is confirmed through simulations. This arises from the fact that applying a magnetic field changes both the real and imaginary parts of the permittivity tensor, in turn changing optical loss behavior directly in the InAs layer. This can also be understood as the effect of a larger total number of carriers (electrons) responding to the magnetic field and thereby imparting the gyrotropic effect to TM radiation. A more in-depth analysis of the thickness dependence and ordering of the carrier concentration is provided later (Fig. S4 and S7).

**Fig. 3:**
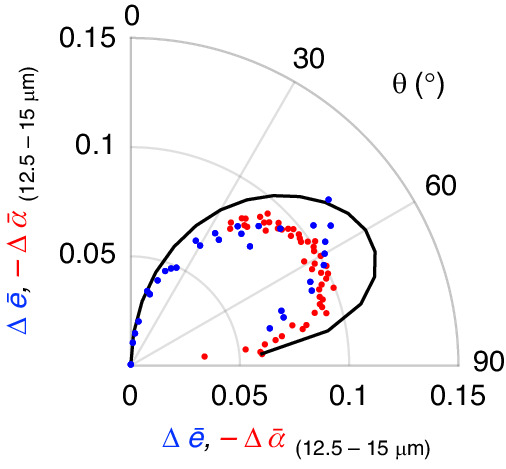
Polar plot of the average change in the emissivity and absorptivity from – 1 T to + 1 T for a spectral bandwidth of 12.5–15 μm. The experimentally measured absorptivity and emissivity are plotted as scatter points (red and blue, respectively) and the simulation results are plotted as a solid black line. The average tuning is largest around θ = 60° for the absorptivity measurements and simulated change in emissivity, in line with the direction of peak emissivity for zero applied magnetic field. While in theory the absolute change in emissivity and absorptivity for the same field should overlap, the emissivity setup has a larger angular uncertainty. A further discussion is included in the Supplement. Note that we plot the negative change in absorptivity to keep all plots on the same axis

The constant spectral position of the broadband Berreman mode across all angles allows us to compare the average change in the absorptivity and emissivity across all measured angles for a fixed spectral range without losing information to angular dispersion of resonances (Fig. S3). Figure 3 plots the average change in the emissivity and absorptivity over a constant spectral bandwidth of 12.5–15 μm when the magnetic field is changed from – 1 T to + 1 T (Δ *ē*_(12.5 – 15 μm)_ and Δ *ā*_(12.5 – 15 μm)_). The scatter points are the experimentally measured change in the emissivity (blue) and absorptivity (red) with the simulated change plotted in solid black. Note that we plot the negative value of Δ *ā*_(12.5 – 15 μm)_ to keep the sign of the axes the same for all traces.

Based on simulations, the maximum tuning of the emissivity and absorptivity occurs around θ = 60–65°. This aligns with the angle at which the sample most strongly emits based on the individual layer thickness. Both Δ *ē*_(12.5 – 15 μm)_ and Δ *ā*_(12.5 – 15 μm)_ have approximately the same angular dependence as predicted by simulations. The slight angular divergence of Δ *ē*_(12.5 – 15 μm)_ is attributed to larger angular uncertainty and noise between emissivity measurements when compared to the absorptivity data. In theory, the two should overlap exactly, as the increased emissivity and decreased absorptivity should be directly correlated.

**Fig. 4:**
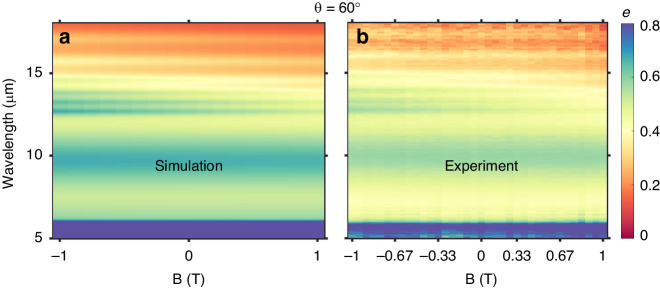
Simulated and measured fine-tuning of emissivity and absorptivity via external magnetic field. Simulation **a** and experimental data **b** showing the emissivity spectra as a function of the applied magnetic field for θ = 60°. We observe stronger tuning of the shorter wavelength modes, corresponding to the layers located nearer the surface of the structure

Going to the angle where the largest Δ *e* and Δ *a* are observed as a function of field (θ = 60°), we performed a fine variation of the magnetic field in both simulation and in our measurements of the emissivity (Fig. 4, Fig. S4). Starting at - 1 T, the individual emissivity peaks from 12.5 μm – 15 μm are visible as green ripples. The broadband Berreman modes of the deeper, lower carrier concentration layers appear as light yellow against the red background of the longer wavelength (λ > 15 μm) emissivity. As we go to zero and positive magnetic field values, the peak emissivity in the 12.5 μm – 15 μm range is strongly tuned from ~0.6 (green) to 0.45 (yellow). This spectral region is tuned stronger than longer wavelengths in large part due to the ordering of the layers (Fig. S5). While it appears that there is no tuning of the emissivity at wavelengths below 12.5 μm, we can resolve a slight tuning of the n^++^ InAs back reflector near 6 μm in the absorptivity measurements and in simulation (Fig. S3). This spectral shift is not resolvable in the direct emissivity measurements due to noise added by atmospheric absorption in this wavelength range.

**Fig. 5:**
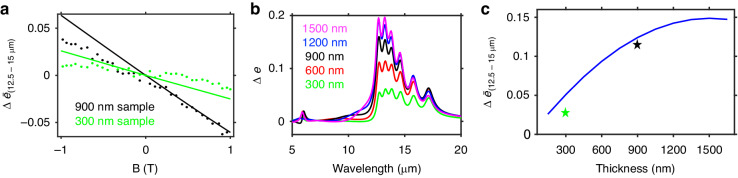
Thickness dependence of the broadband nonreciprocal response. **a** Fine magnetic field dependence of the emissivity from 12.5 μm to 15 μm for a thick (black) and thin (green) sample. The thick sample shows a stronger magnetic field dependence than the thin one. **b** Simulated Δ *e* spectra for varying sample thickness. We observe stronger tuning overall for thicker samples; however the tuning is increasingly skewed blue. Small tuning of the back reflector at 6 μm is also seen in the experimental measurements of the absorptivity (Fig. S3). **c** The average change in the emissivity from 12.5 μm to 15 μm for changing sample thickness. The green and black stars are the experimentally measured values. The angle in this is held at θ = 60°, and it should be noted that changing the thicknesses of the constituent ENZ layers will also influence the directionality

Staying at θ = 60°, we compare the change in the emissivity under external magnetic field for both the thick (150 nm individual layer thickness, 900 nm total thickness) sample and a second, thin (50 nm individual layer thickness, 300 nm total thickness) sample. The tuning of the magnetic field is smaller both in our measurement (scatter) and simulations (solid lines) for the thin sample (green) (Fig. 5(a)). While the angular distribution of the emissivity/absorptivity tends to larger angles for thinner samples^16^, the overall decrease in the magnetic tuning of the absorptivity and emissivity for thinner samples holds true across other angles (Fig. S6).

To explore this thickness dependence in greater detail, we simulate the spectral change in the emissivity (absorptivity) from + 1 T to – 1 T at θ = 60° for samples with linearly varying thickness (Fig. 5b). The thickness of the individual layers is the same, and we use the total thickness when referring to each sample. Two things are discernible when we plot Δ *e* between the two magnetic fields. Firstly, increasing thickness results in larger overall tuning of the emissivity (and absorptivity). Secondly, the spectral distribution of the tuning becomes skewed towards shorter wavelengths within the broadband window. This stronger tuning at shorter wavelengths is a direct result of the ordering of the layers. If one were to place the lowest carrier concentration layer (n_6_) at the top of the structure and reverse the ordering so that the highest carrier concentration layer (n_1_) is situated on the reflector, the tuning at longer wavelengths would exceed the tuning at shorter wavelengths (Fig. S5 and Fig. S8). One limitation of samples comprised of constituent layers with successively increasing thickness is that the directional selectivity of the Berreman mode decreases and the absorptivity and emissivity become more isotropic.

For a configuration where the ENZ gradient goes to higher carrier concentrations (shorter ENZ wavelength) for deeper layers, we note a stronger overall tuning (Fig. S5). This can be understood intuitively: as the free carrier concentration of a layer increases, it can be thought of as more metallic^17,18^. In the case of our sample, having the high free carrier concentration layer on the top means that the emission from deeper layers is absorbed (or absorption of deeper layers is screened by the top layers). This means that re-ordering the carrier concentration gradient not only results in stronger tuning at longer wavelengths, but overall stronger tuning because of lower absorptive losses in the top layers effecting lower layer’s magnetic field response.

To balance a uniform spectral distribution of the difference between emissivity and absorptivity with the overall magnitude of the tuning, we propose a gradient structure with increasing individual layer thickness for the deeper ENZ films (Fig. S7). This increasing thickness with depth offsets the reduced tuning observed for layers located deeper within the sample.

Figure 5c plots the change in the average emissivity at θ = 60° between + 1 T and – 1 T for the spectral range from 12.5 μm to 15 μm for varying total thickness (and layer thickness) of the samples. We include the experimentally measured values as symbols on the plot. While the average tuning increases with increasing sample (and layer) thickness, the effect saturates above 1500 nm total thickness (250 nm individual layer thickness). For these thicker samples, the angle of optimal coupling for the Berreman mode shifts closer to normal incidence. This results in weaker Berreman mode coupling to free-space and could account for the saturation of the effect in the limit of large thicknesses.

## Discussion

We directly measure both the emissivity and absorptivity of a magneto-optically active gradient-ENZ structure that has reduced absorptivity and enhanced emissivity for – 1 T (and vice-versa for + 1 T) magnetic biasing. The effect is broadband and occurs over a wide range of angles. The angle where maximal tuning occurs aligns with the angle of maximum emissivity/absorptivity of the sample when zero magnetic field is applied.

The spectral dependence of the tuning is related to the depth of the layer associated with the wavelength being tuned. In the devices we measured, this meant that shorter wavelengths were tuned more strongly than longer wavelengths due to layers with higher carrier concentrations being located nearer to the top of the sample. Furthermore, the magnitude of the tuning is dependent on sample thickness, with thicker layers resulting in a larger magnitude of tuning. This larger overall magnitude in tuning comes at the cost of uniformity in the spectral distribution of the tuning. To balance this, we put forward a gradient-ENZ structure where the individual layer thicknesses are also varied along the depth dimension as a potential solution.

The general design principles regarding thickness and ordering are applicable to other materials that should exhibit the nonreciprocal absorptivity and emissivity both with (e.g., InSb^19^, graphene^20^) and without a magnetic field (Weyl semimetals^21–23^). A limitation in using 2D materials lies in the thickness requirement of a Berreman mode. Other designs that rely on drift effects^24–26^, and spatiotemporal modulation^4,27^) need to be developed further for broadband nonreciprocal absorptivity and emissivity demonstrations at practical wavelengths. The spectral bandwidth of the nonreciprocal emissivity and absorptivity for this configuration can be tuned by the free carrier concentration, albeit with a reduction in tuning magnitude at shorter wavelengths due to larger scattering rates for higher carrier concentrations. In a fundamental optical mode point of view, methods that allow the heavily doped InAs reflector to effectively push the fields into the gradient ENZ layer and minimize the optical power lost towards the bottom of the emitter would allow more photons to interact with the free carriers, hence enhancing the average contrast. This can be done by increasing the thickness of the heavily doped InAs layer or increasing the doping concentration of the heavily doped InAs layer and thereby having this layer feature a larger, negative real part of the permittivity. In a materials point of view, using a higher moblity, lower electron effective mass material like InSb should enhance the magnetic field response of the structure. Another avenue to increasing the contrast between the nonreciprocal emissivity and absorptivity is to introduce a lossless dielectric spacer layer between the gradient-ENZ layers and the back reflector^28^. By adding such a layer, the phase difference for the absorptivity and emissivity when a magnetic field is applied can result in near-complete destructive or constructive interference.

Demonstrating both nonreciprocal thermal emissivity and absorptivity in an engineerable broadband spectral range that overlaps with the atmospheric transparency window is an important step in bridging theoretical models of nonreciprocal photonic energy conversion^29^ and radiative cooling^30^ to real-world implementations. We hope this work provides a platform for future work to build spectrally tailored, directionally selective nonreciprocal emitters with near unity differences in emissivity and absorptivity. In writing this paper, we became aware of a similar work that measured nonreciprocal absorptivity from gradient-ENZ structures in the far-infrared^9^.

## Materials and methods

### Sample fabrication

Molecular beam epitaxy (MBE) is used to grow the designed InAs-nanolayers with different n-type (Si) doping concentrations. The solid-source VEECO Gen-930 MBE is equipped with a valve-controlled arsenic cracker to provide the As_2_ flux and a 400 cc SUMO cell for indium is operated with a higher tip temperature to reduce the defect density caused by indium spitting. The InAs-nanolayers are grown on epi-ready, single-side polished, semi-insulating GaAs (100) substrates. An oxide desorption process is carried out for the GaAs (100) substrates prior to the InAs-nanolayer growth under an As_2_ flux of 5 × 10^-6 ^Torr at a substrate temperature of 600 °C. A 50 nm GaAs buffer layer is grown at a GaAs growth rate of 0.33 mLs^-1^, in which the substrate temperature decreases to 580 °C. After the buffer layer growth, the GaAs substrate is maintained at 600 °C for 10 minutes under an As_2_ flux to smoothen the surface. For the growth of InAs nano-layers, the substrate temperature is decreased to 410 °C, the InAs growth rate is fixed at 0.5 mLs^-1^ and the As/In flux ratio is controlled to be 1.2–1.5. Following the growth of the gradient InAs film, the same As_2_ flux on the surface is maintained until the substrate cools to 325 °C. Finally, the As_2_ flux is stopped and the sample is taken out of the MBE growth chamber at ~ 275 °C. Reflection high energy electron diffraction (RHEED) is utilized to calibrate the GaAs and InAs deposition rates, as well as to calibrate the As/In flux ratio.

### Measurements

The emissivity and absorptivity measurements are taken with two different setups. The emissivity measurement requires heating the sample above ambient so that the relative emission from the surroundings is sufficiently small^31^. The emission from the sample is beamed into a Nicolet iS50 Fourier transform infrared spectrometer (FTIR). The absorptivity measurements are taken using a J. A. Woollam IR-VASE ellipsometer.

### Simulations

Simulations were done using the COMSOL electromagnetic waves finite-difference frequency-domain package. The assumed Drude model parameters are given in Table 1, and we used a high-frequency permittivity constant *ε*_∞_ = 12.3. The simulations for the full angular and spectral absorptivity of the structure are shown in Fig. S1c.

### Supplementary information

Supplemental Material
