# Supplementary material for: Broadband nonreciprocal thermal emissivity and absorptivity
Source: Light Sci Appl. 2024 Jul 24;13:176. doi: 10.1038/s41377-024-01520-3 (PMC11269630; doi:10.1038/s41377-024-01520-3)
Supplement: Supplementary file 1 — Supplemental Material [file 41377_2024_1520_MOESM1_ESM.docx]

Supplementary Information for

Broadband nonreciprocal thermal emissivity and absorptivity

Komron J. Shayegan,^1,2^ Jae Seung Hwang,^3^ Bo Zhao,^4^ Aaswath Raman, ^3,*^ Harry A. Atwater, ^1,*^

^1^Thomas J. Watson Laboratory of Applied Physics, California Institute of Technology, Pasadena, CA 91125, USA.

^2^Department of Electrical Engineering, California Institute of Technology, Pasadena, CA 91125, USA.

^3^Department of Materials Science and Engineering, University of California, Los Angeles, Los Angeles, CA 90024, USA.

^4^Department of Mechanical Engineering, University of Houston, Houston, TX 77204, USA.

These authors contributed equally: Komron J. Shayegan and Jae S. Hwang

*aaswath@ucla.edu

[*haa@caltech.edu](mailto:*haa@caltech.edu)

**Content**

Emissivity and absorptivity measurement setups

Design considerations: thickness and ordering

**Figures**

**Fig. S1:** Full angular data for emissivity, absorptivity, and simulated values of the sample’s absorptivity with zero applied magnetic field.

**Fig. S2:** Simulated and measured emissivity and absorptivity spectra of sample under magnetic biasing for a range of angles.

**Fig. S3:** Spectral directional change in the emissivity and absorptivity going from + 1 T to – 1 T.

**Fig. S4:** Spectral directional change in the emissivity for a range of magnetic field values.

**Fig. S5:** Spectral dependence of nonreciprocal effect on the ordering of the constituent doped semiconducting layers.

**Fig. S6:** Nonreciprocal directionality dependence on sample thickness.

**Fig. S7:** Gradient-thickness sample to achieve spectrally uniform nonreciprocal absorptivity/emissivity.

**Emissivity and absorptivity measurement setups**

To control the temperature of the sample, we use a TC-48-20 thermoelectric temperature controller set at 100 °C. The heater is covered in aluminum to mask any emission from the heater itself, and the sample is affixed to the heater using Polyonics, Inc. XT-654 double-sided tape (stable up to 300 °C). The sample and heater sit at the axis of rotation of a motorized goniometer cradle (Newport BGS80PP) that can cover an angular range of +/- 45°. The rotation point is sufficiently far (57.5 mm) from the cradle surface that the sample can be between the pole pieces of the electromagnet while the cradle is outside the fringing field of the magnet. We obtain the emissivity of the sample by subtracting out the emission of aluminum over all angles and dividing by the obtained spectrum of our reference blackbody (carbon black deposited onto a Si wafer with carbon paper bonded to it). We found that this provides a dense, uniform, and temperature-stable reference when compared to depositing/combusting directly onto a Si wafer. All measurements were taken at 100 °C.

We use a low numerical aperture zinc selenide lens (25.4 mm diameter, 75 mm focal length) to collect the emission from the samples. This both boosts the signal-to-noise ratio and ensures that we are collecting the emission from the sample (or aluminum, or blackbody reference). One drawback is that it introduces some angular uncertainty when compared to the absorptivity measurement system, with the angle of acceptance being ~ +/- 9.6°. The transmission window of the ZnSe lens also reduces signal strength beyond 16 μm (Fig. S1a). The collected emission is then beamed into Fourier-transform infrared (FTIR) spectrometer (Nicolet iS50). The spectrometer detector is an uncooled deuterated lanthanum triglycine sulfate (DLaTGS) detector. The sensitivity of the detector beyond 16 μm is reduced, resulting in lower signal-to-noise ratio at longer wavelengths.

The absorptivity measurement system is a J. A. Woollam IR-VASE ellipsometer operated in reflection/transmission mode. The IR-VASE uses a SiC globar source in conjunction with a deuterated lanthanum triglycine sulfate (DLaTGS) detector to obtain an interferogram of the reflected light. For the zero-field measurement, we use a gold reference of the same area as the sample itself for a baseline measurement through all angles (35° < θ < 75°). We then collect the reflection data of the sample through all angles and normalize the collected reflection intensity by the reflection intensity of the gold reference. This accounts for the beam spread of the infrared spot at oblique angles and gives us the reflectivity of the sample. The absorptivity is obtained by subtracting the reflectivity from 1 as there is no transmission through the sample (Fig. S1b). The resolution of both the absorptivity and emissivity measurements is 4 cm^-1^. The fast-Fourier transform for both measurements is performed by the internal software of the respective setups (Omnic for emissivity and WVASE for absorptivity).

We apply the magnetic field in our absorptivity setup using a Halbach array [^[[1]](#endnote-1)^], which requires a separate set of stages to align the sample and ensure it is both normal to the source and detector as well as eucentric. Because of a slight occlusion of the beam path by the focusing pieces of the Halbach array, we re-normalize the data to the maxima and minima of the zero-field data (i.e., ranging from 5 μm to 20 μm). Alternatively, one could retake the baseline with the gold reference loaded in the Halbach array. However, given the absence of magneto-optic tuning of the minima and maxima for this sample (i.e., at 5 μm and 20 μm) and the strong agreement with simulations, we find this approach to be sufficient (Fig. S2a, c, f, d, and g).

The emissivity setup uses an electromagnet (GMW 5403) with 38 mm diameter pole pieces spaced approximately 20 mm apart. For both the electromagnet and the permanent magnet, we use a Lakeshore HGT-1010 to measure the field applied across the sample. While the electromagnet and permanent magnet are both capable of going above 1.0 T, we use fields for which the Hall sensor is linear (up to 1.0 T). Defining the angle of observation (θ) relative to the applied magnetic field (*B*) the same as in out absorptivity setup, we get enhanced broadband emissivity for negative magnetic fields and suppressed emissivity for positive magnetic fields. This effect is observed across all angles (Fig. S2b, e, h), however is weakest at near-normal angles (Fig. S2b).

Stitching together the full angular data acquired for the emissivity and absorptivity under external magnetic fields, we see that the intensity of the broadband tuning is dependent on angle, however the spectral location of the tuning exhibits a weak angular dependence (Fig. S3). This is attributed to the flat dispersion relation of the Berreman mode.

At short wavelengths (~ 6 μm), we see a slight tuning of the n^++^ InAs back reflector (Fig. S3b and c). The magnitude of this unintentional tuning is weak for two reasons. The first is that the higher carrier concentration of the back reflector means there is a higher scattering rate, Γ, and therefore smaller off-diagonal component. The second is that the reflector layer is located at the bottom of the structure, with the layers above absorbing most of the outgoing radiation from the back reflector. We leave the comprehensive consideration of design tradeoffs for wavelength of operation with III-V ENZ structures and magnitude of tuning to a future study.

The use of an electromagnet in the emissivity setup allows us to access intermediary magnetic fields. When acquiring the full angular data, we swept the magnetic field at each angle before proceeding to the next angle. This is done instead of maintaining a static field and sweeping through all angles to avoid any hysteresis in the goniometer introducing angular differences between magnetic field measurements. For low magnetic fields (+/- 0.33 T), we see faint tuning of the Berreman modes (Fig. S4c). For high magnetic fields (+/- 1 T), there is strong tuning (Fig. S4a) that is consistent with both absorptivity and simulations (Fig. S3).

As expected, at intermediate fields (+/- 0.67 T), we see the tuning of the Berreman modes with a weaker amplitude than for the high field case (Fig. S4b). We also see something that is an artifact of the measurement setup: blue strips alternating for every other angle measured. This is likely due to a small pull on the sample holder placed between the pole pieces. At low fields, there is little pull; at high fields, the change in magnitude of the Berreman mode as a function of field obscures this “pulling” effect.

**Design considerations: thickness and ordering**

For our structure, the spectral tuning of the emissivity is skewed towards shorter wavelengths because the higher carrier concentration layers are located at the top of the structure and the lower carrier concentration layers located at the bottom are screened by the upper layers (n_1_ 🡪 n_6_). If one were to reverse the ordering of the carrier concentration gradient (n_6_ 🡪 n_1_), one would see the opposite spectral dependence (Fig. S5). Not only would the tuning at longer wavelengths be larger, but the overall tuning magnitude would be larger. Intuitively this can be interpreted as the lower carrier concentration layers behaving less “metallic” than the higher carrier concentration layers, and thus do not screen the deeper layers as effectively.

In addition to the ordering of the gradient-ENZ layers, the thickness of the individual layers also plays a key role in the magnitude, angular, and spectral distribution of the tuning. Experimentally, we measure two samples with different individual layer thicknesses (50 nm and 150 nm) and thus total thickness (300 nm and 900 nm respectively). The two samples had the same carrier concentration gradient. The different thicknesses of the two samples means that even at no applied magnetic field, the angular distribution of the absorptivity/emissivity is different between the two samples. The thinner the sample, the larger the angle of the maximum absorptivity/emissivity [^[[2]](#endnote-2)^]. This aligns with the angle at which the strongest magnetic tuning of the absorptivity occurs (Fig. S6, green trace), however the overall tuning is still weaker over almost all angles over the same spectral bandwidth (12.5 μm – 15 μm).

As discussed in the main text (Fig. 5 (a)), the spectral distribution of the tuning is dependent on the thickness of the sample. For samples with thinner layers, the spectral distribution is more uniformly distributed across all wavelengths associated with the ENZ layers due to lower absorption by layers located near the top of the sample (Fig. S7, green). Conversely, for a sample with thicker individual layers, the tuning for wavelengths associated with the upper ENZ layers increases and while the tuning for deeper layers is suppressed by the upper layers (Fig. S7, black trace).

A way to balance the larger magnitude tuning afforded by thicker layers with the spectral uniformity of the tuning for thinner samples is to introduce a gradient in the thickness of the individual layers. We suggest that the thickness of the individual ENZ layers be increased as the layers go deeper into the sample (Fig. S7, left panel). The thicker layers will have stronger tuning at their respective wavelengths due to the larger electronic contribution/thickness, while the thinner layers located near the top are not as heavily “screened” as thin layers deep in the heterostructure. The resultant tuning is both large in magnitude relative to the thin sample while spectrally more uniform than the thick sample (Fig. S7, blue trace). It should be noted that the intensity of the tuning for the gradient thickness sample could be increased further by swapping the order of the carrier concentration gradient (i.e., going to higher carrier concentrations along the depth dimension). While we plot the spectral tuning for θ = 60°, a deeper analysis of the effect of having a gradient in the thickness layer needs to be done.

To confirm that the screening effect is in part due to the metallic nature of the upper layers, we run simulations for the difference in emissivity from +1 T to -1 T for varying carrier concentration of only the top layer (Fig. S8). We observe that as the carrier concentration of the top layer increases (i.e. becomes more metallic), the tuning effect of the magnetic field is reduced for the deeper, lower carrier concentration layers.


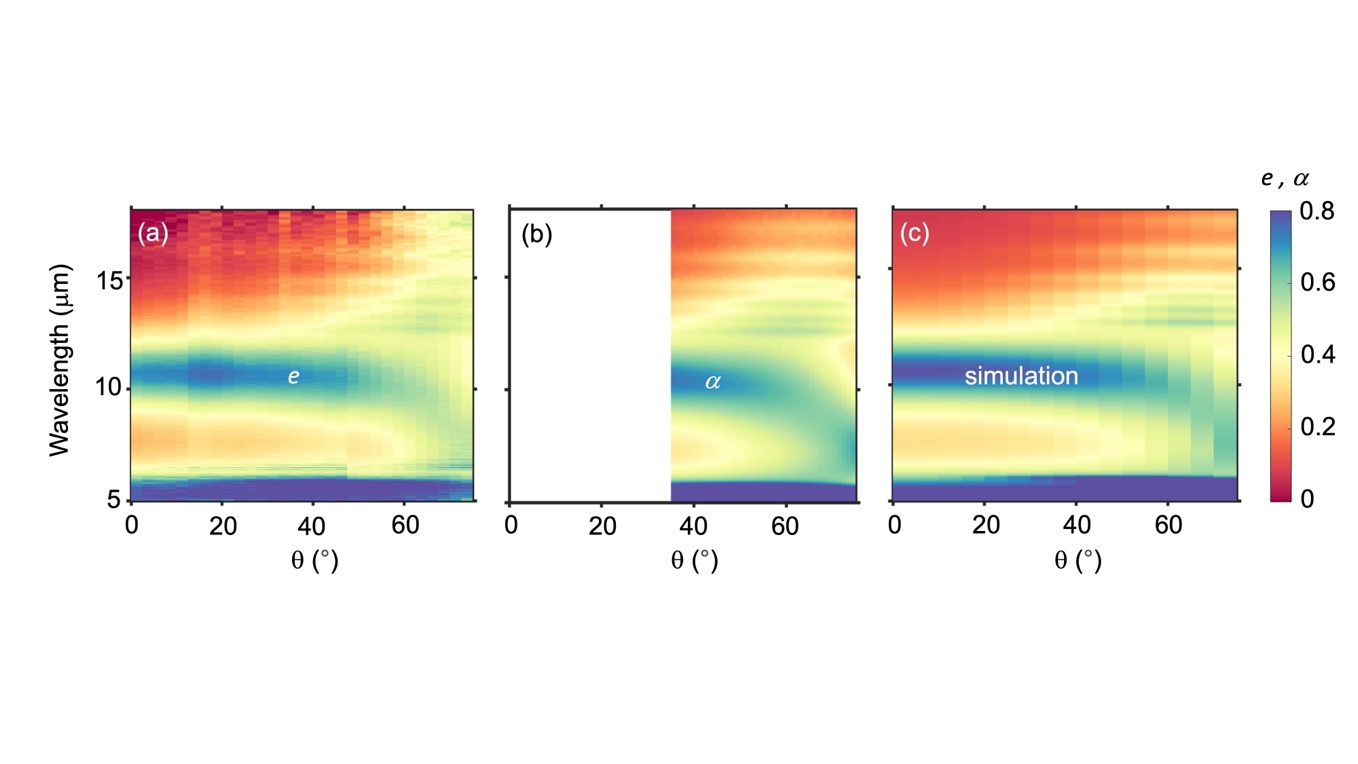


**Fig. S1** (**a)** Direct emissivity at 100 °C, (**b**) absorptivity at 25 °C, and (**c**) simulated spectral directional *e* and *a* from the 900 nm thick structure when zero magnetic field is applied.


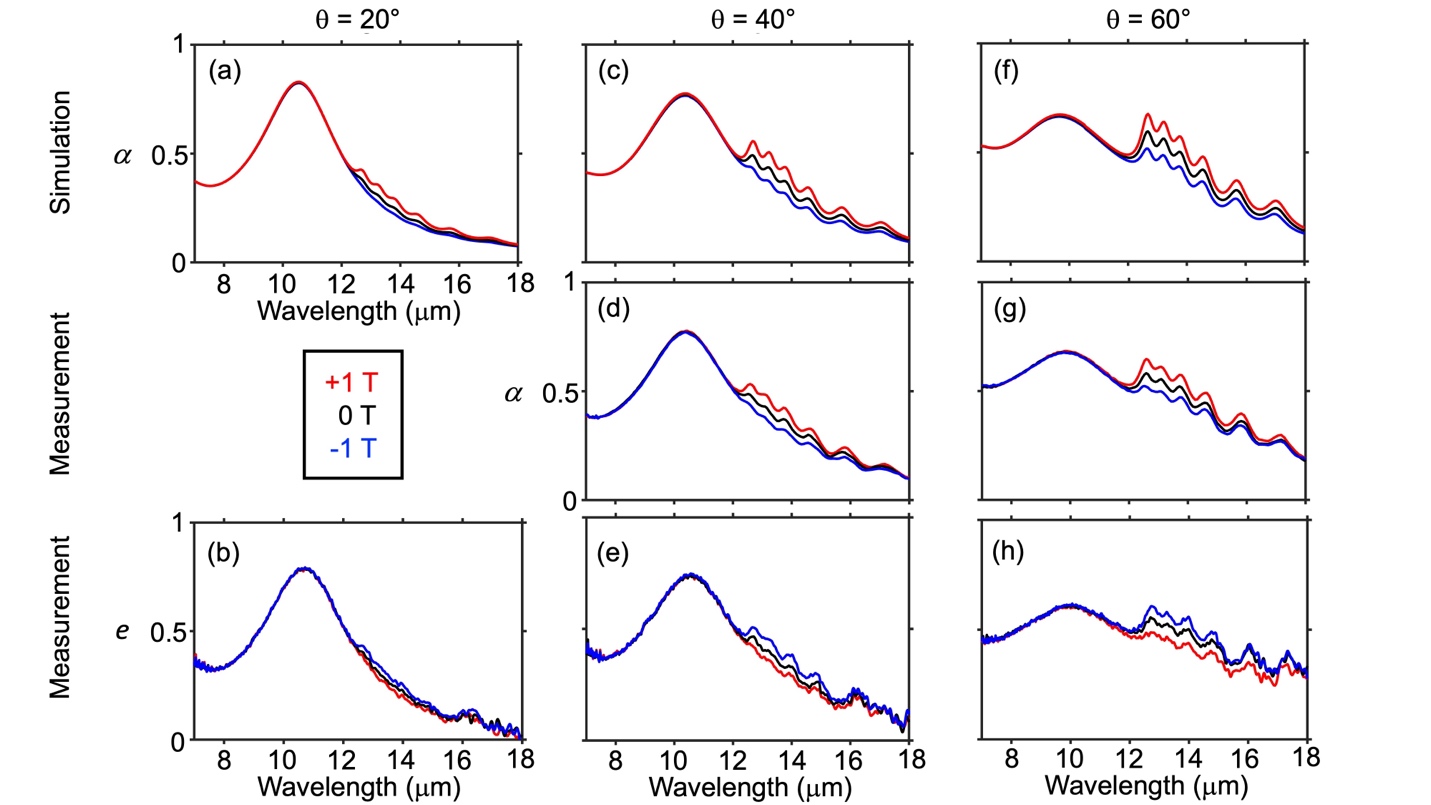


**Fig. S2** Simulated absorptivity, measured absorptivity, and measured emissivity spectra under an external magnetic field for θ = 20° (**a** and **b**), θ = 40° (**c** – **e**), and θ = 60° (**f** – **h**). The absorptivity data is taken at 25 °C and the emissivity data is taken at 100 °C. We note that for wavelengths above 16 μm, the emissivity data increases in noise due to decreased detector sensitivity and transmissivity of the ZnSe lens.


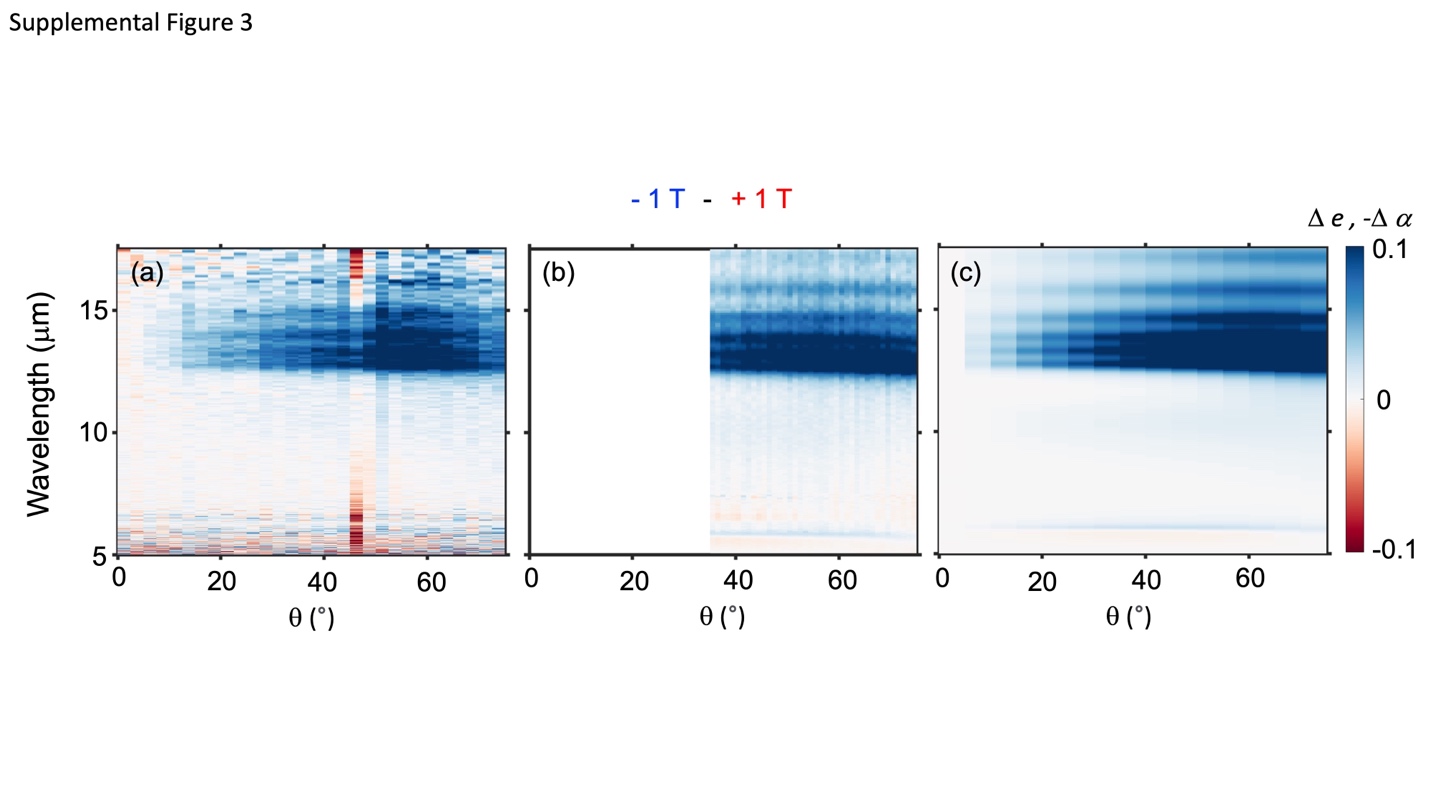


**Fig. S3** Spectral directional change in the emissivity (**a**) and absorptivity (**b**) going from + 1 T to – 1 T. We plot the negative change in the absorptivity to keep the colors of the plots consistent. In simulation (**c**) we also observe a faint strip near 6 μm that corresponds to the n^++^ InAs back reflector of the structure. This is also visible in (**b**), however the noise at short wavelengths in the emissivity measurement obscures this detail.

**
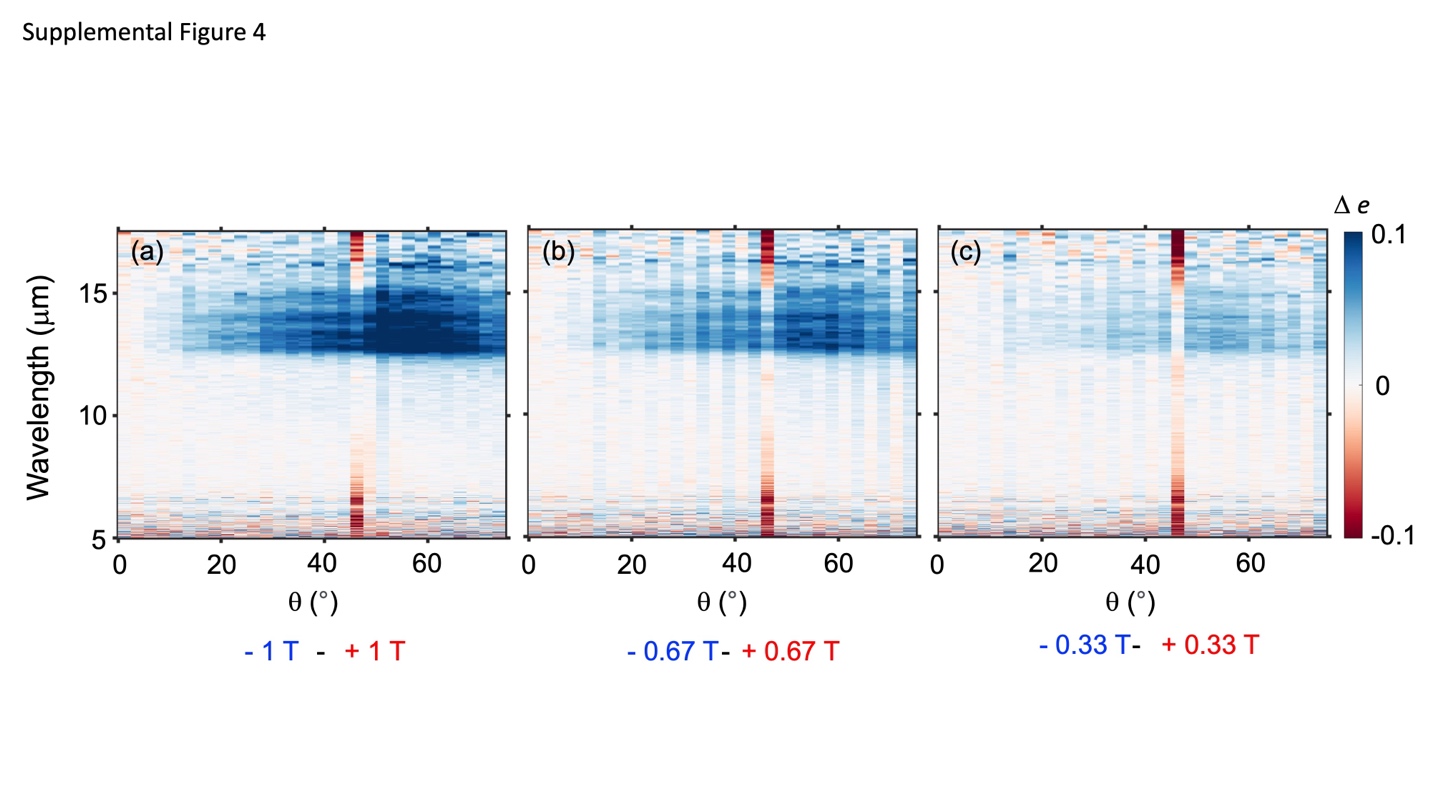
**

**Fig. S4** The spectral directional change in emissivity for the 900 nm structure for (**a**) – 1 T to + 1 T, (**b**) – 0.67 T to + 0.67 T, and (**c**) – 0.33 T to + 0.33 T. We observe a strong magnetic field dependence across 10° < θ < 75°. The red line at short and long wavelengths for θ = 45°is a result of a pause and continuation in the measurement. We attribute the alternating blue stripes visible in the lower-field measurements to a slight pulling effect on the sample stage that is only noticeable when the change in emissivity is weak (i.e., at lower magnetic field strengths).


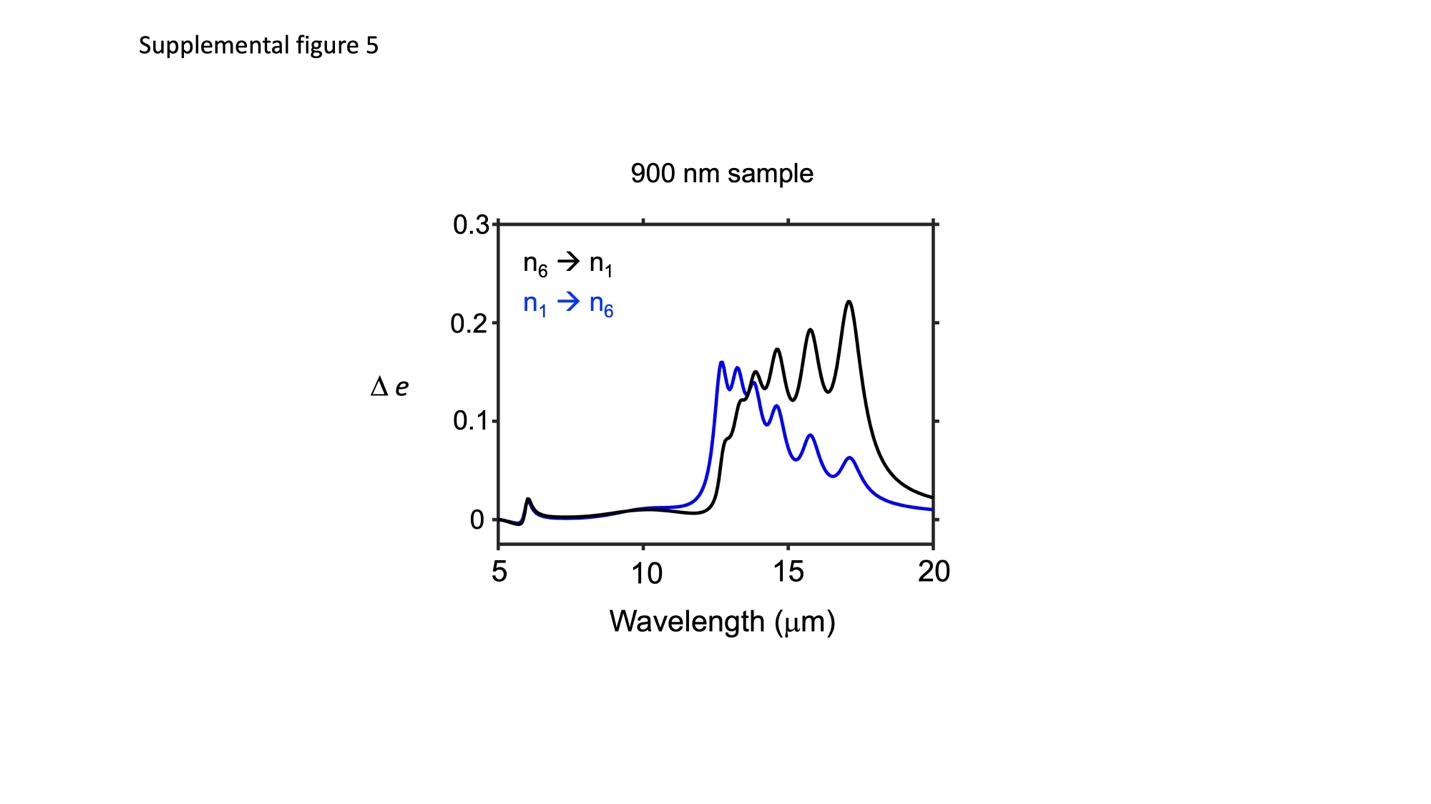


**Fig. S5** Simulated change in the spectral emissivity from – 1 T to + 1 T for the 900 nm sample at θ = 60°. The black trace is for increasing carrier concentration for deeper layers; the blue trace is for decreasing carrier concentration for deeper layers (the sample discussed and experimentally measured in the paper). Reversing the carrier concentration order of the gradient layers results in a reversal in the spectral dependence of the tuning and an overall increase in the effect of the magnetic field on the emissivity and absorptivity.


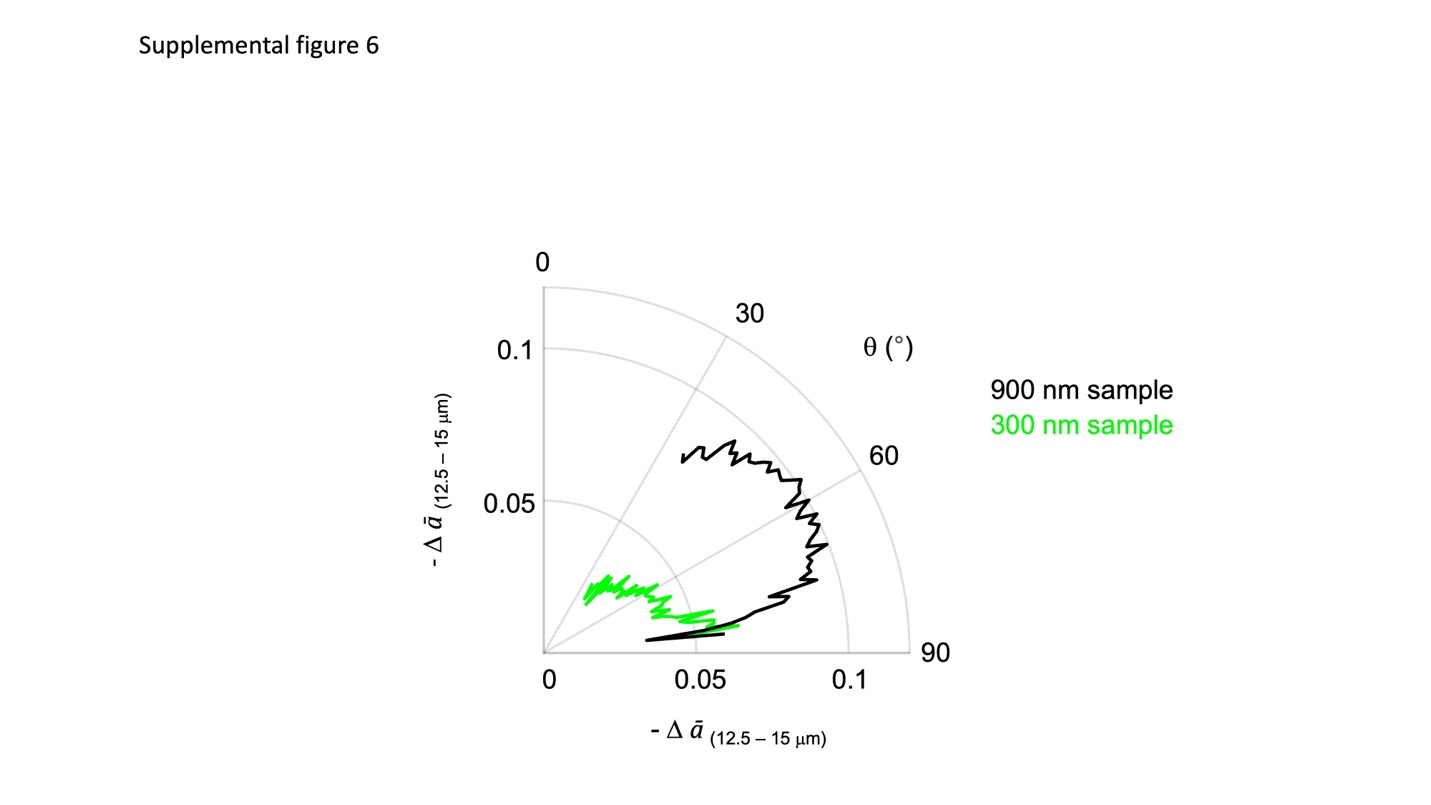


**Fig. S6** Average change in the absorptivity from – 1 T to + 1 T for a thin (green) and thick (black) sample where the carrier concentration gradient is the same. The spectral range of the average is from 12.5 μm to 15 μm. The thin sample’s peak absorptivity and emissivity are at larger angles due to the thickness dependence of the angular dispersion of the Berreman modes. We observe stronger tuning over a wide angular range for the thicker sample.


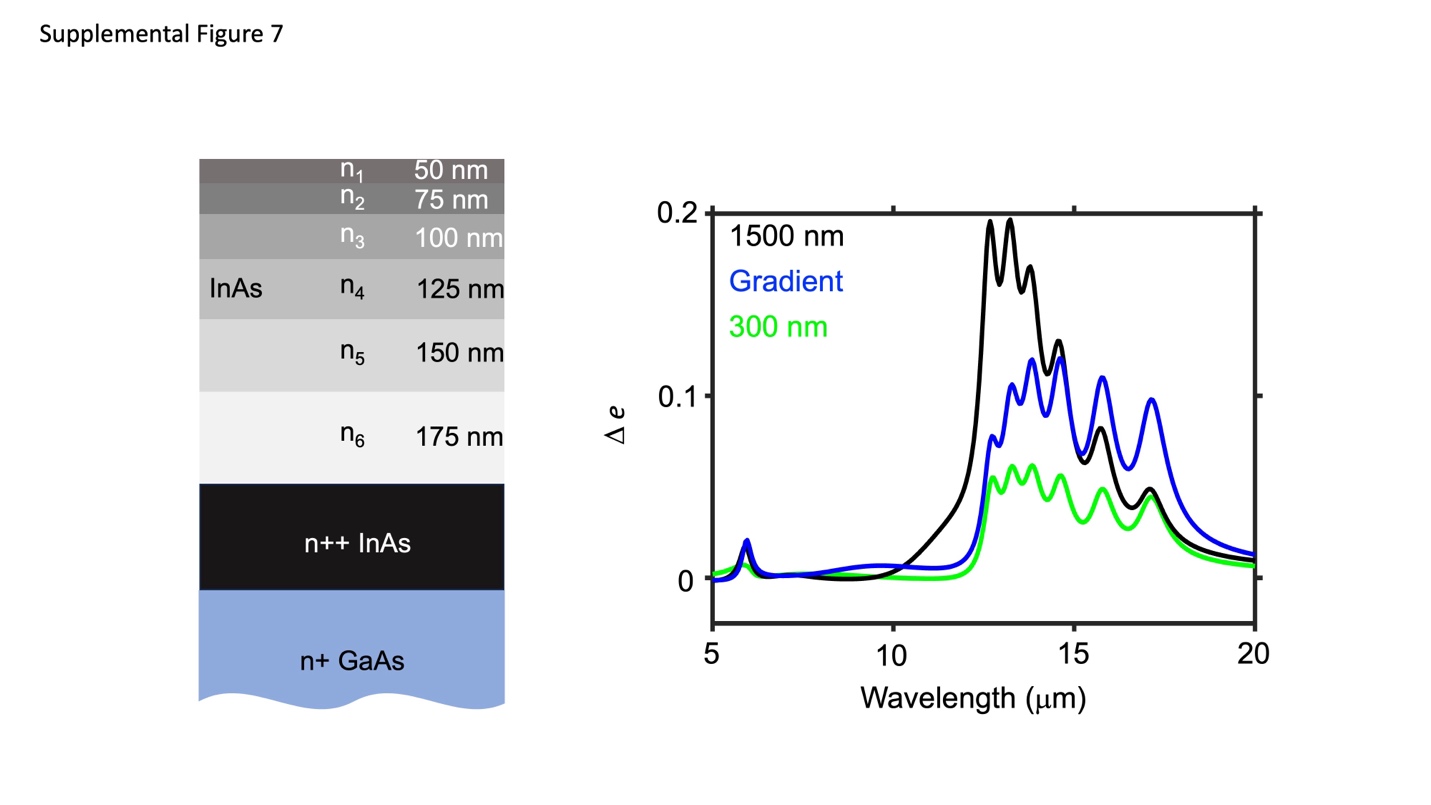


**Fig. S7** Structure with both a gradient in the carrier concentration as well as the thickness (left). The thinner layers on top result in weaker tuning of the wavelengths associated with those layers. However, this also means that the tuning of longer wavelength (deeper) layers is not screened as strongly. The simulated change in the spectral emissivity from – 1 T to + 1 T for θ = 60° show this tradeoff.


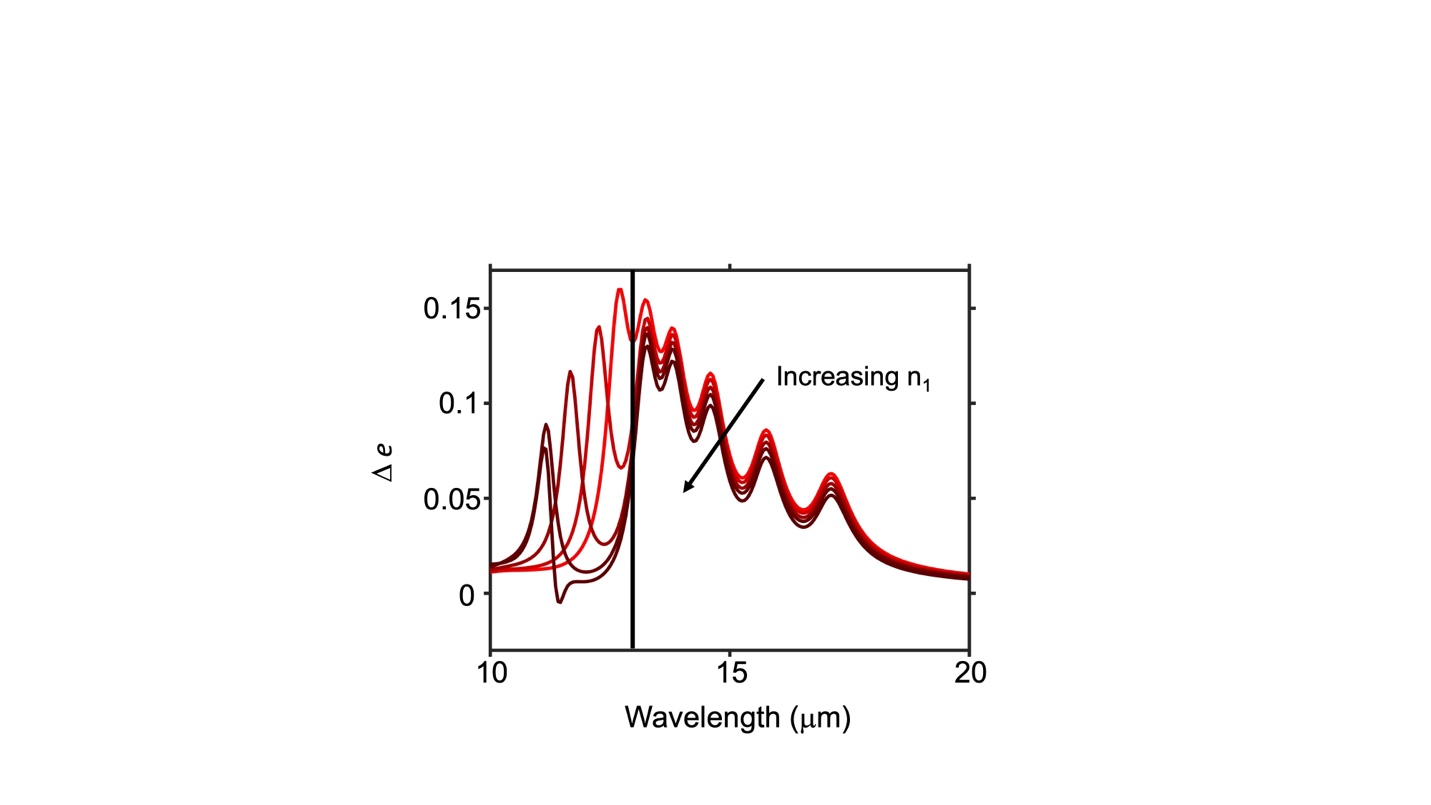


**Fig. S8** Average change in the absorptivity from – 1 T to + 1 T for increasing top layer carrier concentration. As the concentration of the top layer increases (light red to dark red), the tuning of the lower layers is also reduced. The carrier concentrations for the top layer used in the above figure are tabulated in the figure below.

| n_1_ (x10^18^ cm^-3^) | *τ* (THz) | *m*_e_ (9.1 x 10^-31^ kg) |
| --- | --- | --- |
| 4.5 | 5.5653 | 0.053 |
| 5.0 | 5.656 | 0.055 |
| 5.5 | 5.659 | 0.057 |
| 6.0 | 5.661 | 0.059 |
| 6.5 | 5.664 | 0.062 |

**References**

1. [] C. Adambukulam, *et al.*, “An ultra-stable 1.5 T permanent magnet assembly for qubit experiments at cryogenic temperatures,” Rev. of Sci. Inst. **92**(8), 1-9 (2021). [↑](#endnote-ref-1)
2. [] J. Xu, J. Mandal, and A. P. Raman, “Broadband directional control of thermal emission,” Science **372**(6540), 393-397 (2021). [↑](#endnote-ref-2)
